# Social and Economic Burden Associated With Typhoid Fever in Kathmandu and Surrounding Areas: A Qualitative Study

**DOI:** 10.1093/infdis/jix122

**Published:** 2017-07-29

**Authors:** Linda M Kaljee, Alfred Pach, Denise Garrett, Deepak Bajracharya, Kshitji Karki, Imran Khan

**Affiliations:** 1 Global Health Initiative, Henry Ford Health System, Detroit, Michigan; 2 International Vaccine Institute, Seoul, Korea; 3 Coalition Against Typhoid, Sabin Institute, Washington, District of Columbia; 4 Group for Technical Assistance, Kathmandu, Nepal

**Keywords:** typhoid fever, Nepal, qualitative, healthcare access, treatment, diagnosis

## Abstract

Typhoid fever is a significant contributor to infectious disease mortality and morbidity in low- and middle-income countries, particularly in South Asia. With increasing antimicrobial resistance, commonly used treatments are less effective and risks increase for complications and hospitalizations. During an episode of typhoid fever, households experience multiple social and economic costs that are often undocumented. In the current study, qualitative interview data from Kathmandu and surrounding areas provide important insights into the challenges that affect those who contract typhoid fever and their caregivers, families, and communities, as well as insight into prevention and treatment options for health providers and outreach workers. When considering typhoid fever cases confirmed by blood culture, our data reveal delays in healthcare access, financial and time costs burden on households, and the need to increase health literacy. These data also illustrate the impact of limited laboratory diagnostic equipment and tools on healthcare providers’ abilities to distinguish typhoid fever from other febrile conditions and treatment challenges associated with antimicrobial resistance. In light of these findings, there is an urgent need to identify and implement effective preventive measures including vaccination policies and programs focused on at-risk populations and endemic regions such as Nepal.

Global disease burden estimates of *Salmonella enterica* serovar Typhi range between 19.1 and 20.6 million cases and 200000 and 600000 deaths annually [1, 2]. Approximately 90% of these deaths occur in Asia [3]. In South Asia, it is estimated that 23% of the population (approximately 400 million persons) lives in high-risk conditions for waterborne diseases [4]. While the primary source of *S*. Typhi infection is through contaminated food and water, a number of factors contribute to disease burden in endemic settings [5]. These factors include poor sanitation, contact with carriers, living near bodies of stagnant water, climatic conditions, and socioeconomic contexts (eg, literacy rates, food and water consumption patterns) [6, 7].

Multidrug resistance to first-line agents including ampicillin, trimethoprim-sulfamethoxazole, and chloramphenicol has been followed by reported resistance to fluoroquinolones [8]. Antimicrobial resistance has decreased typhoid fever treatment efficacy and increased treatment cost and risk of complications and death [9, 10]. Hospitalization for typhoid fever occurs in 10%–40% of cases. Complications including gastrointestinal bleeding and intestinal perforation occur in 1%–4% of cases [3, 11].

Typhoid fever is highly endemic in Nepal. In recent studies, municipal water in Kathmandu was found to be contaminated with *S.* Typhi and *Salmonella enterica* serovar Paratyphi A, and exposure to these water sources and socioeconomic status have been identified as risk factors for *S.* Typhi [12, 13]. In a retrospective study in 5 hospitals in Nepal, 1881 cases of typhoid fever were culture confirmed, with 70% of those cases in children aged <15 years [14].

Prevention of typhoid fever requires improved water and sanitation infrastructure in endemic regions, ongoing surveillance, early identification of cases through blood culture and other reliable technologies, and health education and community outreach programs [15–17]. In addition, the World Health Organization has advocated for the vaccination of high-risk population groups in endemic settings and the use of vaccines to control outbreaks [3].

There are limited data regarding the social and economic burdens of typhoid fever on affected households and local challenges in relation to disease diagnosis, treatment, and prevention. The primary objectives of this study are to (1) clarify and contextualize the experiences of households affected by typhoid fever from prediagnosis through treatment and ongoing engagement in preventive practices; (2) provide additional perspectives from healthcare providers and outreach workers regarding the challenges and barriers for diagnosis, treatment, and prevention of typhoid fever; and (3) identify potential avenues for interventions to improve access to care and disease prevention.

## METHODS

### Overview

In August 2015, qualitative interviews were conducted with 22 households (case studies) and 8 physicians. Three focus group discussions were conducted—1 with public health center (PHC) providers and 2 with female community health volunteers (FCHVs) at rural and urban PHCs. In addition, a brief instrument was used to assess household monetary and time costs associated with each disease episode. The study research team included 2 US-based social scientists and the director and staff from a Kathmandu-based nongovernmental organization.

### Research Sites

Research sites included 11 urban and periurban sections in Kathmandu and Patan municipalities and 2 rural towns, Dhulikhel and Banepa, approximately 30 km outside Kathmandu.

### Sampling and Recruitment

Qualitative data sampling strategies require identification of salient dimensions for identification of a representative group of respondents [18]. For the household case studies, these dimensions included blood culture diagnosis within the past 6 months of a permanent household member, location (urban/rural), and demographics of the typhoid fever patient (sex/age). The physician sample included various specialists and private and public clinic– and hospital-based practitioners. Nonphysician medical providers included nurses, medical assistants, and midwives from a single PHC. FCHVs were recruited from 2 additional PHCs. The research team worked with local hospitals to identify and recruit patients or caregivers to the study. Physicians, health professionals, and FCHVs were identified through the local research team.

### Data Instruments

Separate and complementary interview guides were developed for household case studies, physicians, PHC staff, and FCHVs. In addition, household members were asked to complete a social-resource mapping activity and a household cost instrument. The social-resource mapping activity included questions about distances and accessibility of health facilities, food markets, and other resources. The household cost instrument asked about specific monetary costs and time spent for doctor visits, hospital stays, diagnostic tests, prescribed and over-the-counter medications, patient food, transportation to and from health facilities (direct costs), lost work time, lost school attendance, and lost time taking care of household and nonhousehold obligations (indirect costs).

### Data Collection

Household case study data were collected either at the respondent’s house or in a private room at the research office in Kathmandu. Physician data were collected at physicians’ offices, and focus group discussion data were collected at the PHCs. Interviews were conducted in English and Nepali by the US-based consultants and local bilingual research staff. One of the 2 US-based consultants speaks Nepali. Data were digitally recorded.

### Data Management and Analysis

Recordings were transcribed and Nepali interviews were translated into English. A coding dictionary was developed and all data were coded in a qualitative data management program. Data searches were conducted based on key research constructs including disease severity, barriers and facilitators to care, diagnosis and testing, use of medications and treatments, social support networks, and prevention practices. Searched data sets were analyzed for patterns within and across respondent groups. Texts were organized within these patterns and are presented in the Results section. Demographic data were entered into Excel software and household cost data were entered into SPSS 22 software. Descriptive statistics were conducted for demographic data and for reported indirect and direct household monetary and time costs.

### Ethical Considerations

The proposed study was reviewed and approved by the Nepal Health Research Council, government of Nepal. All participants consented and signed a written consent form prior to data collection. Data collectors were trained in research ethics and consenting procedures.

## RESULTS

### Demographics

Of 22 household case study respondents, 12 (55%) were female. Fifty-five percent (12/22) of patients were female and 50% were aged ≥18 years. Seventy-five percent (6/8) of participating physicians were male. All FCHVs were female and 67% (4/6) of PHC providers were female (Table 1).

**Table 1.** Demographic Data for Household Case Studies, Physician Interviews, and Health Provider and Female Community Health Volunteers

Data Source	Category	no./No. (%)
Household case studies (N = 22)	Female sex	12/22 (55%)
	Mean age, y (range)	32 (20–49)
	Employed	12/22 (55%)
	Relationship to patient	9/22 (41%) – self
		9/22 (41%) – parent
		1/22 (4%) – spouse
		3/22 (14%) – other relative
	Urban	15/22 (68%)
	Household patient, female	12/22 (55%)
	Household patient, mean age, y (range)	19 (2.5–38)
Physician interviews (N = 8)	Female sex	2/8 (25%)
	Mean age, y (range)	42.4 (27–65)
	Current employment	1/8 (12.5%) – private hospital
		1/8 (12.5%) – private clinic
		2/8 (25%) private–public hospital
		3/8 (37.5%) PHC – public clinic
		1/8 (12.5%) – Institute of Medicine
	Mean years in current position (range)	8.8 (1.3–32)
Health providers (FGD), public health center (N = 6)	Female	4/6 (67%)
	Mean age, y (range)	41 (23–54)
	Current position	1/6 (16.5%) – staff nurse
		2/6 (33.3%) – community medical assistant
		3/6 (50%) – auxiliary nurse midwife
	Mean years in current position (range)	17.7 (9–32)
FCHVs, urban site (N = 6)	Female	6/6 (100%)
	Mean age, y (range)	37.4 (32–41)
	Education	3/6 (50%) – secondary school not completed
		1/6 (16.6%) – health professional school
		2/6 (33.4%) – university/college
	Mean years in current position (range)	15 (10–18)
FCHVs, rural site (N = 9)	Female	9/9 (100%)
	Mean age, y (range)	46 (33–60)
	Education	1/9 (11.1%) – primary school
		3/9 (33/3%) – secondary school not completed
		3/9 (33.3%) – secondary school completed
		1/9 (11.1%) – health professional school
		1/9 (11.1%) – university/college
	Mean years in current position (range)	14.3 (3–26)

Data are presented as no./No. (%) unless otherwise indicated.

Abbreviations: FCHV, female community health volunteer; FGD, focus group discussion; PHC, public health center.

### Household Monetary and Time Costs

Data on illness episode–related costs were collected from 20 respondents. Included in this sample of 20 respondents were 2 hospital workers who had both insurance and leave time, and did not report any direct or indirect costs. Overall, the mean direct and indirect monetary costs per household per episode were US$92 and US$32, respectively. Mean direct and indirect time costs per household were 10 days and 22 days, respectively. Nine individuals reported loss of income during the illness episode with a mean loss of US$37, and 12 individuals reported a mean loss of 6 work days. Three of the respondents reported borrowing money (US$23–$284) from relatives or an employer. Nine individuals reported a mean loss of 15 school days (range, 1–30 days) and 14 individuals reported a mean loss of 12 days performing routine household tasks (range, 1–28 days). Individuals who were hospitalized reported greater time and monetary costs (Table 2).

**Table 2.** Comparison of Direct and Indirect Monetary and Time Costs Associated With Typhoid Fever Illness Episodes (Hospitalized and Nonhospitalized Cases)

Costs	Hospitalized	Nonhospitalized
Total direct monetary costs^a^	US$166 (SD, US$81)	US$39 (SD, US$38)
Total indirect monetary costs^b^	US$67 (SD, US$104)	US$5 (SD, US$7)
Total direct time costs^c^	20 d (SD, 30 d)	3 d (SD, 4 d)
Total indirect time costs^d^	27 d (SD, 17 d)	18 d (SD, 19 d)

Abbreviations: SD, standard deviation; US$, United States dollars.

^a^Includes provider fees, hospital fees, diagnostic tests, prescription and over-the-counter (OTC) medication costs, purchase of food, and transportation costs.

^b^Includes loss of income or expenses associated with work, school, routine household and nonhousehold duties/tasks.

^c^Includes time for patients and caregivers at providers**’** offices, hospital stay, diagnostic tests, purchasing prescription and OTC medications, purchasing food, and transport.

^d^Includes patients**’** and caregivers**’** loss of time at work, school, and engaging in routine household and nonhousehold duties/tasks.

### Qualitative Household Case Studies, Interviews, and Focus Group Discussions

#### Prediagnosis

Household respondents reported a range of symptoms associated with typhoid fever episodes. In addition to fever, adult patients and caregivers of children and adolescents reported myalgia, headache, lethargy, sweating, poor appetite, vomiting, and stomach pain. Symptoms were often initially interpreted to be a “common cold” or a “seasonal fever.” Household respondents also related the symptoms to specific behaviors such as being out in the sun or bathing in cold water.

*“I thought it was a seasonal fever. He was in Kathmandu only 5 days so it may be due to the weather change …”* (uncle of 16-year-old male patient)

*“I use to take a bath daily in the winter as well as the summer, so she [mother] thought that I had a common cold. She took it [the fever] to be normal, but I didn’t recover…”* (19-year-old female patient)

Initially, all household respondents reported some form of self-treatment during the first 2–4 days after onset of fever. A majority reported use of paracetamol (acetaminophen) to relieve fever and body aches. Only 2 household respondents reported use of antibiotics prior to blood culture diagnosis and one of those respondents was a physician. One respondent reported use of “massage” for a child who was complaining of stomach pain. Decisions to seek medical advice and care were usually associated with increasing symptom severity and/or prolonged fever.

*… [his] fever was more than before and he was complaining that his stomach was burning. Then we decided to take him [to the health facility]…”* (parent of 2.5-year-old male patient)

*“I didn’t care at first [about symptoms] but then I had diarrhea…”* (30-year-old female patient)

The vast majority of household respondents reported going to clinics or hospitals. Two respondents discussed use of traditional healers, and during focus group discussions with FCHVs, there were several comments in relation to traditional medicine integrated with use of biomedicine.

*“At that time no, I didn’t go for a traditional healer. I waited for one village brother [healer], but he was not there, so I went to the hospital … when discharged from the hospital … the fever was under control but the headache was continuing. So I called my neighbor’s brother who is a traditional healer. After he looked at me, after 2 days I was getting relief.”* (49-year-old female patient)

*“When I was with my parents and my relatives saw my child’s sickness. They suggested to go for the traditional healer … I took her for the first time to the traditional healer, [but] immediately the next day I took her to the hospital.”* (parent of 5-year-old female patient)

*“First they go to a local Shaman and if it won’t work then they come to us.”* (rural PHC)

*“One of my sister’s sons, it is really hard to feed him and he gets sick from time to time. So they took him for Jhar Phuk [healing through sweeping and blowing with mantra]. Just today he went to a shaman to the Vaidya [Ayurveda physician]. It’s not because they can’t afford the doctor, but they have a belief towards the traditional treatment.”* (urban PHC)

#### Diagnosis and Treatment

As previously noted, respondents were diagnosed using blood culture, which is considered the gold standard and exhibits greater specificity and sensitivity than more readily available serological tests (eg, Widal) [17]. The majority of the respondents reported that the physician provided immediate treatment for typhoid fever based on his/her clinical diagnosis while waiting for the blood test results. A few respondents reported their health provider was uncertain or “doubtful” that the patient had typhoid fever.

In our interviews with physicians, they discussed challenges of diagnosis for typhoid fever, options available for uncertain diagnoses, and decisions regarding treatment.

*“…whenever we suspect enteric fever, it’s completely based on the clinic findings like high grade fever … a patient complaining they have been having the fever for the past 4 or 5 days and they have been taking paracetamol but it’s not working … I suspect those cases if [the patient] doesn’t have chest involvement or if they don’t seem to have urinary tract infection … and it is a high grade fever, I strongly suspect enteric fever.”* (physician, rural PHC)

*“… your experience says it is typhoid, the patient is toxic. Can you leave this patient without giving any medications, any antibiotic? I prefer to prescribe some broad spectrum antibiotic…”* (physician, urban hospital)

A majority of respondents living in urban settings had a choice of possible medical facilities and generally few barriers related to distance. Alternatively, some rural respondents did discuss logistical barriers and complicated traveling routes.

*“We got help from a friend to go to Panauti. From Panauti, we took a local bus, then we took another bus to Dhulikhel. But while traveling I was so sick my husband called the ambulance and we went to the hospital in an ambulance.”* (17-year-old female patient)

While services and medications at PHCs are free or only require a small fee, for some households, even a minimum charge is more than they can afford. In addition, nontreatment costs such as travel can be expensive. FCHVs also discussed low literacy combined with poor economic conditions as barriers to seeking healthcare.

*“There are a few who cannot even pay the minimal amount of 10 Rs in our clinic for the registration … they say that they had forgotten [the money] and they do not return …”* (FCHV, urban PHC)

*“… sometimes they come here and get an antibiotic for a few hundred rupees but then they pay more money just travelling and staying at a hotel…”* (physician, rural private–public hospital)

*“And the reason behind late diagnosis is that the people in our community are illiterate. So, they take a paracetamol and start working when they have a fever … it is also because of their low economic status.”* (FCHV, rural PHC)

While the choice of health facility is often made based on cost, other variables such as previous experiences at a facility, and trust in the quality of services and personnel, also affect decisions. Parents also express need to seek the best care for their children despite costs.

*“…it’s a private hospital. I went there because I trust that hospital.”* (uncle of 16-year-old male patient)

*“…when children get sick, parents do not get concerned with money. Typhoid is a very dangerous disease and needs more money and medicine.”* (parent of 13-year-old female patient)

Care and treatment of patients required extensive amounts of time and effort, both from household members and extended social networks. A vast majority of household respondents described support from family, as well as neighbors and friends.

*“My husband took care [of me] for 5 days and for 2 days my daughter’s husband’s brother’s wife who is working as a hospital staff nurse [helped us].”* (49-year-old female patient)

*“His mother took care of the child at the hospital … at night my husband and me both took care of him and at day myself. One day I had to close my shop because my baby was scared by the intravenous injection … my mother-in-law was at home and she helped to cook food and to wash the clothes.”* (parent of a male patient [toddler; age not reported])

The importance of a strong social network was also discussed in an FCHV group.

*“There are lots of difficulties when someone in the family is not feeling well. One of the members give all of his time to take care of the sick. If that person goes to study, then his studies are hampered. If he is a job holder, then he might lose his job … so there might be economical difficulties … whenever there is a sick person in the house then it is difficult for the whole family.”* (rural PHC)

#### Postillness Care and Prevention

Length of illness varied significantly across respondents, with a few individuals reporting acute symptoms for a week while others up to a month. Many respondents reported longer-term effects including continued fatigue, poor appetite, and headaches. Respondents also discussed concerns about recurrence of typhoid fever. These individuals described ongoing care through use of supplements and special diets.

*“In our experience it is serious … [the] doctor told us to care for her diet. I called to her hostel and requested for daal bhat [rice and lentils] only with lots of liquid … this disease is difficult as it needs more time and care.”* (parent of 13-year-old female patient)

*“…now her father has bought chyabanprash [a nutritional supplement] to overcome her weakness…”* (parent of 16-year-old female patient)

Respondents were asked about engagement in preventive practices after their experiences with typhoid fever. About a quarter of respondents said that they had not made any changes in their household habits or activities. For some, this was a consequence of feeling inadequately informed about the causes of typhoid fever. Others expressed concerns about conditions in their communities beyond their control.

*“Now I am cleaning my room, kitchen, toilet, and I am becoming hygienic too. I also suggested to my friend to maintain his personal hygiene.”* (21-year-old male patient)

*“We were concerned about water. We have a good filtering system for drinking water. But most probably he was suffering by drinking water from school.”* (parent of 7-year-old male patient)

*“We don’t know the preventing measures. We know that one may be suffering from typhoid when they have fever, so I wish we never have fever…”* (parent of 17-year-old female patient)

FCHVs are actively engaged in prevention activities. Other sources of health information come from physicians and nursing student programs, as well as school-based health education efforts.

*“We have the nursing students here, who regularly educate the patients particularly on infectious diseases and how to prevent diseases … so, we do have education programs in the ward as well as in the out-patient department … we try to go to the schools and then educate the young children …”* (physician, rural private–public hospital)

*“It is because of laziness that they do not boil water”; “Maybe it is because they have not understood”; “One is the economic reason. They do not have gas so it’s difficult to boil”; “And another thing, is since a long time people drink water without boiling and so they say now why should we boil”; “Some do not boil water because they do not like the taste of boiled water…”* (FCHVs, rural PHC)

Discussions regarding use of the currently available typhoid Vi polysaccharide vaccine were generally positive both among household respondents and physicians. However, only a few household respondents were aware that a typhoid fever vaccine is available on the market. There were also some caveats regarding use of a vaccine including physician recommendations, vaccine efficacy, and potential adverse events.

*“Various [typhoid fever] vaccines have come, some injectable, some oral capsules, but they were not effective very frankly up till now…”* (physician, urban public hospital)

*“I didn’t hear about typhoid vaccine. If there is no side effect than I will take the typhoid vaccine. No matter it is 400 or 1000 rupees there should not be side effect…”* (uncle of 16-year-old male patient)

## DISCUSSION

The Nepali healthcare system is a complex integrated pluralistic system of public and private services including biomedicine, Western and traditional pharmaceuticals, and traditional and spiritual/religious healers [19–21]. Previous research indicates that strategies of health seeking employed by households in response to an acute illness episode often involve multiple and overlapping efforts. Qualitative research can help to elicit these patterns for specific symptoms and diseases, and provide important healthcare utilization data that can be used to improve surveillance activities and in development of interventions to decrease mortality and morbidity [22, 23].

In the current study, data indicate that the nonspecific symptoms of typhoid fever not only result in delay in households seeking outside medical treatment, but also present diagnostic challenges for physicians. During initial disease onset, respondents did not associate their symptoms with typhoid fever. Within households, use of common Western pharmaceuticals for reduction of pain and fever is an early response to a typhoid fever episode. Early treatments may be combined with use of traditional medications or treatments, though relatively few household case study respondents discussed use of traditional methods. Early symptoms including fever may be attributed to “folk etiologies” such as exposure to too much sun or bathing in cold weather. These data are similar to research conducted on typhoid fever in Zanzibar, in which respondents discussed traditional religious beliefs about the causes of their symptoms, and also combined use of Western and traditional medicines during onset of the disease [23].

When symptoms continued and/or increased in severity, household respondents described their decision to seek outside medical care. Several factors contributed to the choice of facility. These included convenience (eg, distance, ease of transport), cost, experience and trust, and availability of physicians. These findings are similar to a study conducted on healthcare utilization in Pakistan in which the primary criteria for selecting a clinic/hospital were proximity, familiarity with the provider, and responsivity of the staff [24].

A previous cost-of-illness study conducted in Asia indicates a range of costs for care between countries. The lowest cost for nonhospitalized cases was US$13 in Kolkata, India, and the highest cost was US$67 in Hechi, China. Costs associated with hospitalized cases ranged from US$129 in Kolkata to US$432 in North Jakarta, Indonesia [25]. Although the current research did not include an actual cost-of-illness study, our data on monetary and time costs incurred during the typhoid fever episodes are similar to those reported costs in countries in the region and indicate a significant burden both in terms of time and money. The combined direct and indirect mean monetary costs for hospitalized patients was US$233—just slightly under a third of the Nepali gross national income (US$730). In terms of time, hospitalization compared to home care required greater number of days for direct care and greater loss of days for work, schooling, and household obligations. With increasing antimicrobial resistance of typhoid fever strains, these monetary and time costs can be assumed to be increasing as treatment modalities become less effective.

Costs affect not only household members, but also extended family and other support network members who may be asked to lend money and/or provide assistance with routine household tasks. Also important to note are the longer-term costs for some households in relation to providing special foods and supplements for individuals even after acute symptoms have subsided. These data support research on the role of strong social networks as related to access to health resources and to subsequent health outcomes [26, 27].

Prevention strategies and resources are focused in multiple settings including hospitals and clinics, schools, and the community. A number of barriers decrease the effectiveness of these programs including low literacy rates, fuel costs, routine practices, and perceived “taste” of boiled water. Even within households experiencing typhoid fever, almost 25% of respondents had not made changes to decrease future risk.

Data indicate that typhoid fever vaccination is feasible, cost-effective, and efficacious, particularly in endemic settings; however, to date there remains limited market-based use of typhoid fever vaccines in Nepal [28]. Overall, respondents were positive in relation to future use of a typhoid vaccine, although some physicians remained skeptical about the utility of current vaccines. Generally, few household respondents were aware that typhoid fever vaccines exist and are to a limited degree available in Nepal. While policy and programmatic efforts are under way to facilitate introduction of typhoid fever vaccines in endemic areas, other preventive programming needs to be strengthened to both decrease current risk and supplement future vaccination public health programs. As typhoid fever vaccine introduction moves forward, prevaccination campaigns targeting both the general public and health providers will be essential to successful implementation.

There were limitations to this study. The current study only included 22 households, as a salient dimension for participation was a blood culture diagnosis of typhoid fever within the past 6 months. This is a minority of typhoid fever cases in Nepal, as clinical and/or Widal tests are most commonly used. These data do not include community members who may not use or have access to clinics and hospitals. The study was conducted in Kathmandu and 2 nearby rural towns and therefore is not representative as the vast majority of Nepalese live in rural and often remote communities. To obtain more generalizable data, future research needs to expand beyond blood culture– confirmed cases and can benefit from a combined hospital-/clinic-based and community-focused recruitment strategy.

## CONCLUSIONS

The current study provides important contextual data on the impact of typhoid fever on households in Kathmandu and surrounding areas. Disease burden remains high in Nepal, and significant social, economic, and physical costs from typhoid fever affect households and communities throughout the country. Physicians face challenges related to availability of diagnostic tools and increasing antimicrobial resistance. Further research on the social and economic burdens of typhoid fever in Nepal and other endemic settings is needed to supplement ongoing surveillance, cost-of-illness studies, and vaccine demonstration projects and ensure that household and community experiences are part of future public policies, and treatment and prevention efforts.

## Notes

***Disclaimer.*** The funding agencies had no role in data collection or writing the manuscript.

***Financial support.*** L. M. K. and A. P. have received funding from the Coalition Against Typhoid Secretariat, a program sponsored by the Bill and Melinda Gates Foundation (award number OPP1017518).

***Potential conflicts of interest.*** All authors: No reported conflicts of interest. All authors have submitted the ICMJE Form for Disclosure of Potential Conflicts of Interest. Conflicts that the editors consider relevant to the content of the manuscript have been disclosed.
